# Inhibitory Effect of Eco-Friendly Naturally Synthesized Silver Nanoparticles from the Leaf Extract of Medicinal *Detarium microcarpum* Plant on Pancreatic and Cervical Cancer Cells

**DOI:** 10.31557/APJCP.2020.21.5.1247

**Published:** 2020-05

**Authors:** Ismail Abiola Adebayo, Hasni Arsad, Haladu Ali Gagman, Noor Zafirah Ismail, Mohammed Razip Samian

**Affiliations:** 1 *Integrative Medicine Cluster, Advanced Medical and Dental Institute, Universiti Sains Malaysia, 13200 Bertam, Kepala Batas, Malaysia. *; 2 *Microbiology and Immunology Department, School of Biomedical Sciences, Kampala International University, Western Campus, Ishaka-Bushenyi, Uganda. *; 3 *School of Biological Sciences, Universiti Sains Malaysia, 11800 USM, Pulau Pinang, Malaysia. *; 4 *Department of Biological Sciences, Faculty of Sciences, Bauchi State University Gadau, 751 Itas Gadau, Nigeria. *

**Keywords:** Silver nanoparticles, Detarium microcarpum, pancreatic cancer, cervical cancer, inhibitory activity

## Abstract

**Background:**

Recently, nanoparticle synthesis by eco-friendly methods has received tremendous attention due to the method advantages and also because of the application of the nanoparticles in cancer research. Therefore, in this study, we synthesized silver nanoparticles from *Detarium microcarpum* leaf phytochemicals and evaluated its inhibitory effect on pancreatic and cervical cancer cells.

**Materials and Methods:**

Silver nanoparticles (dAgNps) were synthesized by reacting phytochemicals of *D. microcarpum *leaves with silver nitrate for 12 hours. Cell viability assay was carried out to investigate the cytotoxic effect of dAgNps on HeLa and PANC-1 cells.

**Results:**

Scanning electron microscopy (SEM) and transmission electron microscopy(TEM) results revealed the average sizes of dAgNps are 81 nm and 84 nm respectively. The x-ray diffraction (XRD) pattern of dAgNps was similar to that of face centered cubic(fcc) structure of silver as reported by joint committee on powder diffraction standards (JCPDS) and fourier-transform infrared spectroscopy (FTIR) analysis showed that some phytochemicals of *D. microcarpum *such as polyphenols and flavonoids were likely involved in the reduction of Ag+ to form nanoparticles. Finally, cell viability assay revealed dAgNps inhibited PANC-1 and HeLa cell proliferations with IC_50_ values of 84 and 31.5 µg/ml respectively.

**Conclusion:**

In conclusion, the synthesized nanoparticles from *D. microcarpum *leaves (dAgNps) have inhibitory effect on pancreatic and cervical cancer cells.

## Introduction

Nanotechnology is one of the most important research fields that involve the synthesis of nanoparticles from gold, silver, and iron with sizes that are within the range of 1 – 100 nm (Ng et al., 2012; Mittal et al., 2013; Muthoosamy et al., 2015; Allafchian et al., 2016; Salari et al., 2016). This research field has received tremendous attention from researchers because of the many applications of the synthesized nanomaterials in medicine, telecommunication, engineering, and textile industry, to mention but a few (Hanan et al., 2018). In Medicine, nanoparticles are used in drug development and delivery. The nanoparticles are used for the delivery of drug to a specific target in cells, tissues or organs. Nanoparticles can as well be used for infectious disease and cancer therapy (Thakkar et al., 2010; Hanan et al., 2018). There are many physical and chemical methods that are used for metallic nanoparticle synthesis, which include heat evaporation, electrochemical reduction, lithography, photochemical method, and radiation method (Balashanmugam and Kalaichelvan, 2015; Chandran et al., 2016). These methods often successfully synthesize desired metallic nanoparticles in larger quantities with specific shapes and sizes within relatively short durations (Allafchian et al., 2016). However, these methods require toxic chemical solutions and they produce hazardous waste products that have negative impacts on natural environment and human health (Salari et al., 2016). Also, some of these methods are not economically favourable because they are highly expensive (Allafchian et al., 2016). Therefore, there is a need for an eco-friendly and cheaper alternative method for nanoparticle synthesis.

Natural sources such as plants, bacteria, and fungi are now being used in recent times for the synthesis of nanoparticles in a method called biological synthesis. Unlike other methods, this method simply involves the reduction reaction between electron-rich chemical compounds of plants/microorganisms and the metallic ions to form nanoparticles (Singh et al., 2016). Silver nanoparticles which were reported to possess antibacterial and antiproliferative activities on cancer cells have been synthesized from extracts of different plants such as Pedalium murex (Anandalakshmi et al., 2016), *Urtica dioica* (Jyoti et al., 2016), *Clerodendron serratum* (Raman et al., 2015), *Alternanthera sessilis *(Firdhouse and Lalitha, 2013), and *Erythrina suberosa *(Mohanta et al., 2017).

*D. microcarpum *is an underutilized medicinal plant that is grown in most African countries. It is culturally used for treatment of diabetes, malaria, syphilis, leprosy, diarrhoea, sore throat, etc. (Okolo et al., 2012). The plant is highly rich in electron donating phytochemical compounds such as flavonoids, polyphenols, diterpenes and polysaccharides (Labulo et al., 2016). It is also reported that *D. microcarpum *has strong antioxidant capacity to scavenge free radicals such as intracellular ROS of cancer cells (Adebayo et al., 2019; Lamien-Meda et al., 2008; Rouamba et al., 2017). In this study, we synthesized silver nanoparticles using the extract of Detarium microcarpumleaves and the cytotoxic effects of the synthesized nanoparticles (dAgNPs) on pancreatic (PANC-1) and cervical (HeLa) cancer cells were evaluated for the first time.

## Materials and Methods

*Plant collection, authentication, and phytochemical extraction*

Detarium microcarpum leaves were retrieved from its tree in the forest of Bauchi state of Northern Nigeria. The plant was identified and authenticated at the Department of Biological Science, Ahmadu Bello University, Zaria, Nigeria, and a voucher of the plant was deposited at the University Herbarium. The leaves were grounded to powder and the powder (50g) was added to 250 ml of distilled water. The mixture was continuously mixed for 5 consecutive days and the extract was filtered out using Whatman No. 1 filter paper. The extract was then freeze dried. The solid form of the extract was weighed and kept at 4˚C until further use.

*Silver nanoparticle synthesis*

**Figure 1:**
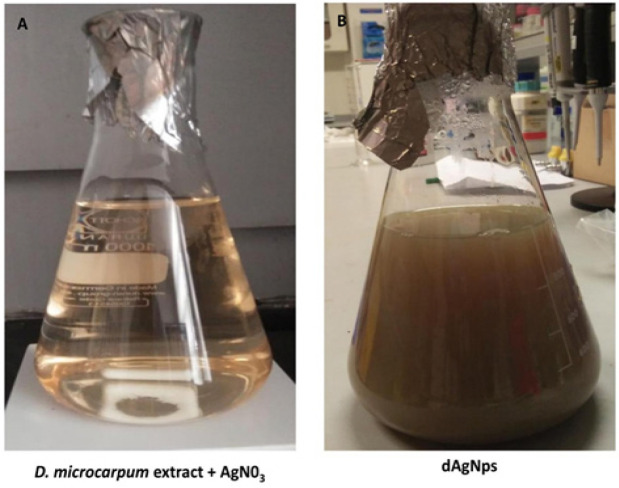
Synthesis of dAgNps before (A) and after (B) the Reaction

The silver nanoparticles (dAgNps) were synthesized using a modified method that was previously reported by Maria et al., (2015). The extract was re-suspended in water and the liquid extract was mixed with silver nitrate in a ratio of 1 (0.5 mg/ml extract): 4 (5 mM AgNO3) (v/v). The reaction was allowed to occur for 12 hours in the dark at 30˚C. The absorbance of the mixture was obtained thrice during the reaction (6, 9, and 12 hours) by UV-Vis spectrophotometer (AK36735, Perkin Elmer, USA) to monitor the synthesis of the silver nanoparticles. A change in colour of the mixture to dark brown signified the end of the reaction after 12 hours (Figure 1). The synthesized dAgNps were recovered by centrifugation of the mixture at 12,000 rpm for 15 minutes. The recovered dAgNps were re-suspended in distilled water and centrifuged thrice to purify them. The purified dAgNps were freeze-dried and kept until further use.

*Characterization of the synthesized dAgNps*

Aside from monitoring the synthetic reaction with UV-vis spectroscopy, we characterized the synthesized dAgNps using Scanning Electron Microscopy (SEM), Transmission Electron Microscopy (TEM), X-Ray Diffraction (XRD) and Fourier-transform infrared spectroscopy (FTIR). For TEM, the solid powder of the silver nanoparticles was suspended in distilled water and a few drops of the suspension were placed on carbon-coated copper grid. The nanoparticles were allowed to deposit and attached to the grid, and then excess solution was blotted off the grid. The grid was allowed to dry prior to the microscopic analysis. SEM was also performed to visualize the shape and measure the size of the dAgNps. XRD was carried out to determine the structural pattern of dAgNps. FTIR analysis was performed using FTIR- Perkin Elmer, USA (2011 model) and data were retrieved within 4,000 – 600 cm-1 wavelengths.

*Cell culture and Maintenance*

Pancreatic cancer cells (PANC-1) and cervical cancer cells (HeLa) were obtained from AddexBio and ATCC respectively and they were cultured and maintained in supplemented RPMI1640 medium (1% penicillin/streptomycin antibiotics, 10% Foetal Bovine Serum) in a CO_2_ saturated incubator at 30˚C.

*Cell proliferation assay*

The cell proliferation assay was carried out using Presto Blue reagent to evaluate the cytotoxic effect of dAgNps on the cancer cells. 5,000 cells/ 100 µl were seeded in each well of 96-well plate for 24 hours. The medium was discarded and replaced with dAgNps (0 – 140 µg/ml) or cisplatin (positive control; 0-50 µM) diluted medium. The cells were treated for 72 hours. Then, Presto Blue cell viability reagent (Invitrogen, USA) was applied based on the recommended method by the manufacturer.

## Results

*Characterisation of dAgNps*

**Figure 2:**
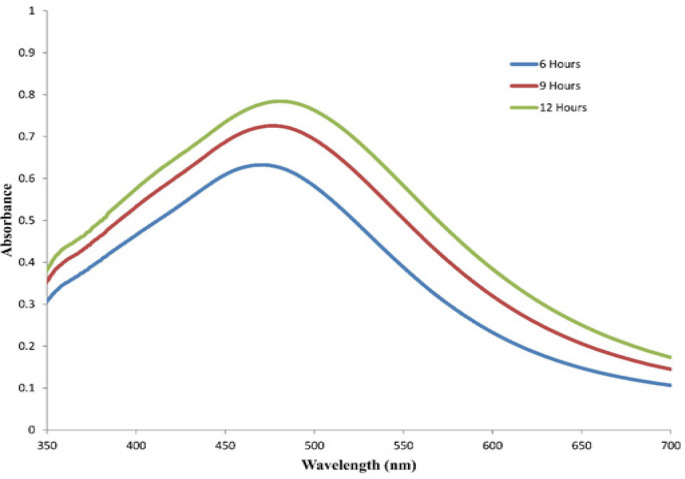
UV-vis Spectrophotometry of the Synthesized dAgNps

The synthesis of dAgNps was monitored by UV-vis spectrophotometer. The surface plasm on resonance (SPR) peak was detected at around 481 nm which is an indication that silver nanoparticles were synthesized (Figure 2). The SPR peak was wide which meant that the sizes of the nanoparticles are not uniform. It was observed that the Surface Plasmon Resonance (SPR) peak of the reaction mixture increased with time and optimum synthesis was achieved at 12 hours. These preliminary observations revealed that the formed dAgNps are well dispersed and they are of different sizes. 

*SEM and TEM*

**Figure 3:**
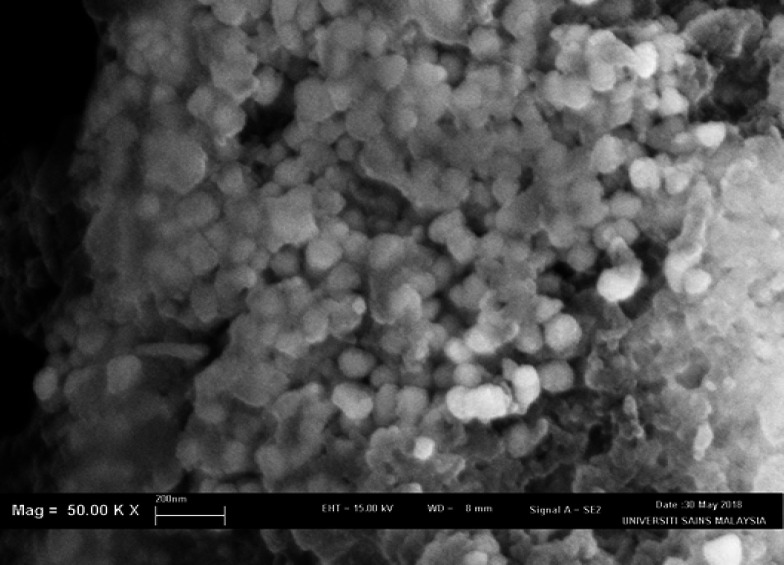
SEM Image Showing dAgNps Morphology

**Figure 4:**
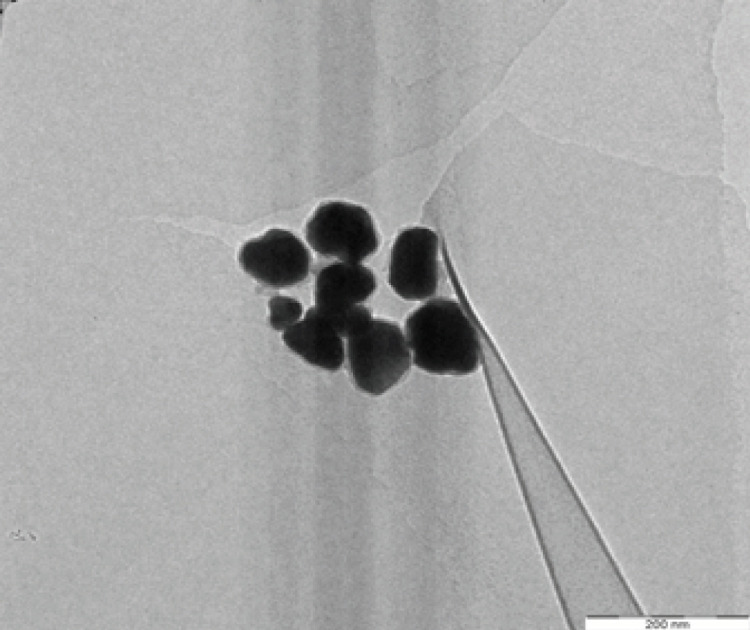
TEM Image Showing dAgNps Morphology

SEM and TEM were performed to confirm the sizes of dAgNps and visualize their shapes. Both microscopic analyses revealed that dAgNps have different shapes and sizes. The images showed that dAgNps were polydispersed with different sizes and most of the nanoparticles were spherical, while others have circular and rectangular shapes (Figures 3 and 4). SEM revealed the sizes of dAgNps were within 62 - 102 nm and the average size is 81 nm. TEM revealed the nanoparticle sizes were within 62 – 103 nm with the average size of 84 nm.

*XRD*

**Figure 5:**
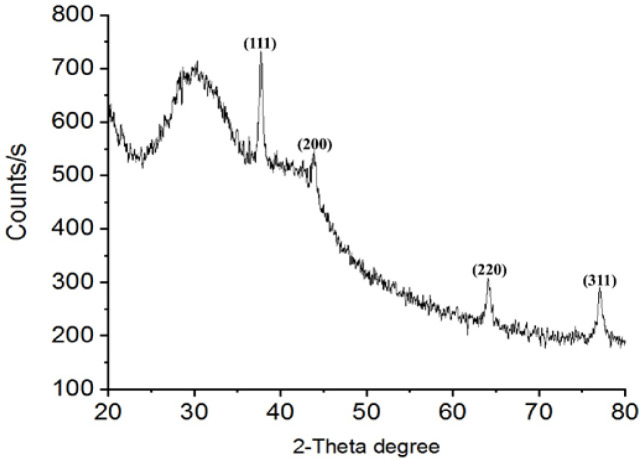
XRD Pattern of dAgNps

XRD of dAgNps was also carried out. The XRD pattern of the synthesized dAgNps was observed to determine if they have crystallized face-centred cubic structure similar to that of silver. The results (Figure 5) showed that the particles have intense peaks at around Bragg’s reflection angles (2θ); 380, 44.080, 64.610, and 77.040 degrees. The calculated interplanar spacing values (d values) of dAgNps for 111, 200, 220, and 311 planes were 2.384, 2.053, 1.441 and 1.237 respectively.

*FTIR*

**Figure 6:**
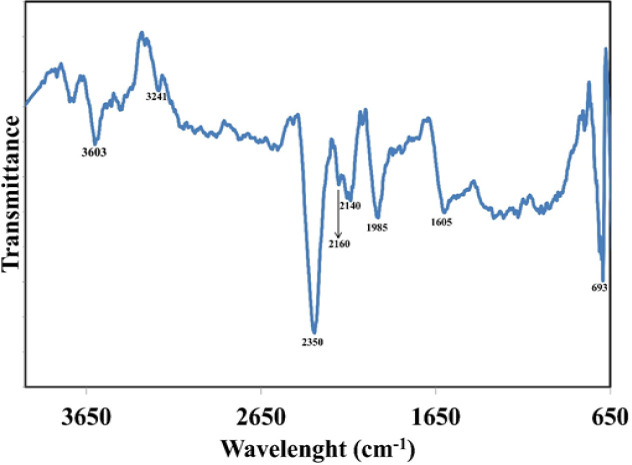
FTIR Spectra of dAgNps

FTIR analysis of the synthesized dAgNps was carried out to identify the possible functional groups of the phytochemical compounds of *D. microcarpum *that were involved in the reduction of silver ion by donation of electrons to synthesize the nanoparticles (Figure 6). Six prominent absorption peaks surfaced at around 3,603, 2,350, 2,140, 1,985, 1,605 and 693 cm^-1^. Other peaks appeared at 3,241 and 2160 cm^-1^. 

*Inhibitory activity of dSNPs on cancer cells*

**Figure 7:**
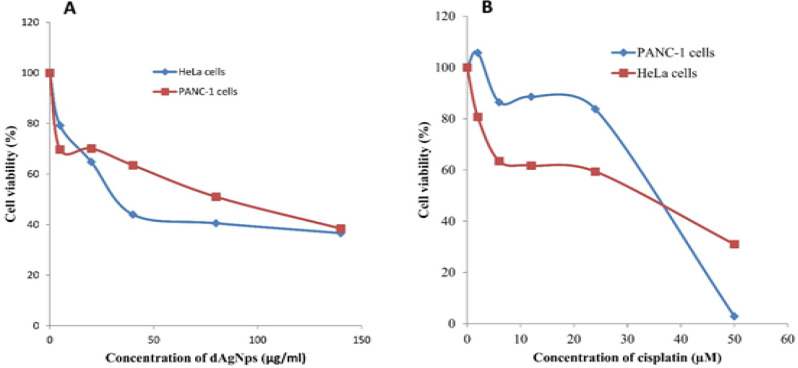
Cell Viability assay of Cancer Cells Treated with dAgNps (A) and Cisplatin (B)

The results of the Presto Blue cell viability assay revealed that dAgNPs inhibited the proliferation of Panc-1 and HeLa cancer cells in a concentration dependent manner (Figure 7). The IC_50_ concentrations of its inhibitory effect on PANC-1 and HeLa cell proliferation were 84 and 31.5 µg/ml respectively (Table 1). A broad spectrum anticancer drug, cisplatin that was used as positive control had IC_50_ values of 33 and 35.6 µg/ml for the inhibition of PANC-1 and HeLa cell proliferations respectively (Table 1). 

**Table 1:** IC_50_ Cytotoxicity Concentration of dAgNps and Cisplatin

Cancer cells	dAgNps (µg/ml)	Cisplatin (µM)
PANC-1	84	33
HeLa	31.5	35.6

## Discussion

Green synthesis of nanoparticles is the preferred method of nanoparticle production because it poses no threat to human wellbeing and the environment unlike the other methods. Plant phytochemicals are part of the natural sources that are used in the green synthesis of silver nanoparticles. In this study, the aqueous extract of Detarium microcarpum leaves was used to synthesize silver nanoparticles (dAgNps). dAgNps were characterized and their inhibitory effect on HeLa and PANC-1 cancer cells was evaluated. The estimated average sizes of dAgNps from SEM (81 nm) and TEM (84 nm) results were within the ideal size range of silver nanoparticles (1 – 100 nm). The XRD results (Figure 5) showed that the particles have intense peaks at around Bragg’s reflection angles (2θ); 380, 44.080, 64.610, and 77.040 degrees which correspond to 111, 200, 220, and 311 lattice planes respectively. More so, the calculated interplanar spacing values (d values) for 111, 200, 220, and 311 planes were 2.384, 2.053, 1.441 and 1.237 respectively. Hence, the synthesized dAgNps have typical face-centered cubic structures similar to that of the silver that was reported by JCPDS committee (file no. 89-3722) (Kokila et al., 2015; Jyoti et al., 2016).

FTIR analysis revealed the absorption peaks of dAgNps. The absorption peak at 3603 cm-1 can be attributed to the stretching of free hydroxyl group or OH group attached to polyphenols and alcohol (Mohanta et al., 2017). The peak at 2350 cm-1 corresponds to -C≡C- stretch (Jain and Mehata, 2017). The peak at 1985 cm-1 can be attributed to –C=O- stretch of carbonyl group (Ali et al., 2015; Gomathi et al., 2017). The observed peak at 1605 cm-1 signifies the presence of –C=C- which indicates the involvement of the alkene groups and their derivatives such as aromatic compounds in nanoparticle synthesis (Mohanta et al., 2017). The smaller peaks that appeared below 1,000 cm^-1^ (1,000 – 650 cm^-1^) can be assigned to the vibration of the stretching of –C-H- group of aromatic compounds in the plant (Saif et al., 2016). In general, it is clear that many electron donating compounds of *D. microcarpum *which are majorly phenolic and flavonoid compounds are involved in the synthesis (Meda et al., 2017).

The Presto Blue cell viability results showed that dAgNps effectively inhibited the growth of the cancer cells (Figure 7). Even though, this is the first report on the inhibitory effect of dAgNp on cancer cells as far as we know, our results agree with other reports on plant-mediated nanoparticles that have cytotoxic effects against HeLa and PANC-1 cancer cells (Sukirtha et al., 2012; Baharara et al., 2018; Sarkar and Kotteeswaran, 2018; Zielinska et al., 2018) . The source of the reductants used for nanoparticle synthesis is an important factor that determines its effectiveness. *D. microcarpum *is an underutilized herb that has large amounts of varieties of phenolic and antioxidant chemical compounds (Lamien-Meda et al., 2008). And it is a well-known fact that antioxidants are generally potential anticancer agents due to the ability to scavenge or suppress intracellular ROS, hence, the effectiveness of dAgNps could be linked to the plant phytochemicals (Saeidnia and Abdollahi, 2013; Adebayo et al., 2018).

In conclusion, the synthesized nanoparticles (dAgNps) from Detarium microcarpumleaves have inhibitory effect on pancreatic and cervical cancer cells, hence, dAgNps are potential source of cancer chemotherapy.
